# Persistent candidemia in pediatrics: exploring risk factors

**DOI:** 10.1016/j.jped.2026.101535

**Published:** 2026-03-25

**Authors:** Adriele C. Siqueira, Amanda M.M. Ferreira, Stella C.S.B. Silva, Bianca Sestren, Damaris Krul, Diancarlos P. de Andrade, Saloe D.B. Poubel, Regiane N. Spalanzani, Marinei C. Ricieri, Fábio d.A. Motta, Terezinha I.E. Svidzinski, Luiza S. Rodrigues, Libera M. Dalla-Costa

**Affiliations:** a Instituto de Pesquisa Pelé Pequeno Príncipe, Curitiba, PR, Brazil; b Faculdades Pequeno Príncipe, Curitiba, PR, Brazil; c Hospital Pequeno Príncipe, Curitiba, PR, Brazil; d Universidade Estadual de Maringá, Paraná, PR, Brazil

**Keywords:** Invasive candidiasis, Persistent candidemia, Children, Risk factor, Mortality, Host characteristics

## Abstract

**Objective:**

Persistent candidemia (PC) in children can lead to unfavorable outcomes. However, its risk factors and clinical impact remain poorly understood. This study aimed to identify risk factors associated with PC in pediatric patients.

**Method:**

We conducted a retrospective analysis of 141 children (0–17 years) diagnosed with candidemia at a children’s hospital in Brazil between 2016 and 2022. Clinical data were collected from medical records. Microorganisms were identified by MALDI-TOF MS, tested for biofilm production, and sensitivity profile. Molecular typing was performed on the three most prevalent species, and *ERG11* mutation screening was carried out on fluconazole-resistant isolates.

**Results:**

PC was identified in 34.8% (n = 49) patients. Independent risk factors included early non-removal of the central venous catheter, parenteral nutrition, and cancer. The overall 30-day mortality was 23.4%, and the candidemia-related mortality was 16.3%. *C. parapsilosis* was the prevalent species. All isolates except one produced biofilm. One isolate of *C. tropicalis*, which had the missense mutation Y257H in *ERG*11, was resistant to fluconazole. Isolates showed high genetic diversity.

**Conclusions:**

PC was associated with host factors and clinical management rather than aspects of the etiological agent, highlighting the importance of early patient monitoring.

## Introduction

Globally, *Candida* is the leading cause of invasive fungal infections (IFIs). Although part of the human microbiota, it can become pathogenic in immunocompromised individuals or when defense barriers are disrupted, causing infections from mucocutaneous to invasive disease (invasive candidiasis – IC) [1,2]. ICs are associated with mortality rates in pediatric patients ranging from 10% to 47% [3].

Candidemia is the most common form of IC. Although both adults and children are susceptible to this disease, the epidemiology and risk factors differ between the groups. Within the pediatric population, the highest rates of candidemia are observed in neonates and infants < 1 year of age [3].

Persistent candidemia (PC) is a complication of candidemia, defined by the isolation of *Candida* spp. from sequential blood cultures after the initiation of antifungal therapy [4]. The reported incidence of PC varies widely, from 8% to 93%, largely due to inconsistent definitions, particularly regarding the time threshold used to define persistence, which ranges from 3 to 10 days across studies. A 5-day interval has been widely adopted as a clinically meaningful period to assess microbiological clearance and therapeutic response. Persistence of bloodstream infections is concerning because it is associated with treatment failure and increased mortality [[5], [6], [7]].

Risk factors for PC encompass host- and pathogen-related aspects. However, evidence is largely limited to adults and neonates, with scarce data in pediatric subgroups [4,6]. Clarifying these factors in children may enable early identification of susceptible patients and mitigate adverse outcomes of persistent infection. In this context, the aim of this study was to investigate the risk factors for the occurrence of PC in pediatric patients.

## Methods

### Isolates and clinical setting

This retrospective study included pediatric patients (aged 0 - 17 years) admitted to a tertiary pediatric hospital with 372 beds in southern Brazil. All the patients were diagnosed with candidemia between August 2016 and August 2022. During the study period, 191 candidemia episodes were identified. After applying the exclusion criteria, 141 episodes remained for the final analysis. Supplementary Figure 1 shows the flow diagram of screened cases, exclusions with reasons, and the final study population.

### Definitions

PC was diagnosed when the positive blood cultures were still obtained at ≥ 5 days after starting antifungal treatment [4]. Patients who had a positive blood culture and did not receive antifungal treatment or prophylaxis were excluded as persistence could not be assessed. Patients who died on the same day as blood culture collection were included to assess early mortality potentially related to candidemia. Crude mortality was defined as death within 30 days of a positive blood culture, whereas mortality attributed to candidemia was defined as death within 7 days of a positive blood culture [8].

### Data collection and ethical approval

Clinical and demographic data, including age, sex, hospitalization unit, previous surgery, associated comorbidities, treatment, persistence of infection, and clinical outcome information, were obtained from electronic medical records. This study was approved by the Institutional Review Board (IRB #2.096.359) of the participating hospital in accordance with the guidelines established for the protection of participants.

### Statistical analysis

Statistical analyses were performed using R statistical software version 4.3.2 [9]. Measures of central tendency (mean and median) and dispersion (standard deviation) were used for descriptive analysis of the sample. Pearson's chi-square and Fisher’s exact tests were used to determine the association between categorical variables. Comparisons between independent groups were performed using Student's t-test or Analysis of Variance (ANOVA). To identify independent predictors of persistent candidemia, a multivariable logistic regression analysis was performed. All variables described in Table 1 were included as candidate variables in a stepwise selection process, and only those showing statistical significance (p *<* 0.05) were retained in the final model. Results were expressed as odds ratios (OR) with their respective 95% confidence intervals.

**Table 1:** Univariate comparative analysis of the clinical characteristics of pediatric patients with persistent and non-persistent candidemia.

Variables	Candidemia		
	**Persistent–n (%) / (n = 49)**	**Non-persistent–n (%) / (n = 92)**	*p*-value
Gender	\	\	0.77
Female	26 (36.6)	45 (63.4)	\
Male	23 (32.9)	47 (67.1)	\
Hospital Setting	\	\	0.642
ICU	26 (32.5)	54 (67.5)	\
No ICU	23 (37.7)	38 (62.3)	\
Prior Pathologic Conditions			
Malignancy	\	\	**0.042**
Hematological neoplasia/Solid tumor	14 (53.8)	12 (46.2)	\
No	35 (30.4)	80 (69.6)	\
Bone marrow transplant	\	\	0.714
Yes	2 (25)	6 (75)	\
No	47 (35.3)	86 (64.7)	\
Neutropenia	\	\	0.655
Yes	5 (45.5)	6 (54.5)	\
No	44 (33.8)	86 (66.2)	\
Heart diseases	\	\	0.261
Yes	12 (46.2)	14 (53.8)	\
No	37 (32.2)	78 (67.8)	\
Renal diseases	\	\	0.181
Yes	5 (20.8)	19 (79.2)	\
No	44 (37.6)	73 (62.4)	\
Liver diseases	\	\	0.739
Yes	4 (40)	6 (60)	\
No	45 (34.4)	86 (65.6)	\
Neurological disease	\	\	**0.02**
Yes	9 (20)	36 (80)	\
No	40 (41.7)	56 (58.3)	\
Invasive procedures			
Surgery	\	\	1
Yes	33 (35.1)	61 (64.9)	\
No	16 (34)	31 (66)	\
Abdominal surgery	\	\	0.588
Abdominal	14 (40)	21 (60)	\
Others	19 (32.2)	40 (67.8)	\
CVC	\	\	0.399
Yes	43 (33.3)	86 (66.7)	\
No	6 (50)	6 (50)	\
CVC removal within 5 days of starting treatment	\	\	**0.005**
Yes	17 (23)	57 (77)	\
No	26 (48.1)	28 (51.9)	\
Mechanical ventilation	\	\	0.667
Yes	24 (32.4)	50 (67.6)	\
No	25 (37.3)	42 (62.7)	\
Parenteral nutrition	\	\	**0.033**
Yes	21 (48.8)	22 (51.2)	\
No	28 (28.6)	70 (71.4)	\
Medicines			
Gastroprotective agent	\	\	1
Yes	28 (34.1)	54 (65.9)	\
No	21 (35.6)	38 (64.4)	\
Corticosteroids	\	\	0.187
Yes	17 (27.9)	44 (72.1)	\
No	32 (40)	48 (60)	\
Chemotherapy	\	\	1
Yes	6 (37.5)	10 (62.5)	\
No	43 (34.4)	82 (65.6)	\
Antibiotics	\	\	0.665
Yes	48 (35.6)	87 (64.4)	\
No	1 (16.7)	5 (83.3)	\
Antifungal prophylaxis			0.396
Yes	14 (42.4)	19 (57.6)	\
No	35 (32.4)	73 (67.6)	\
Antifungal administered to treat candidemia	\	\	0.731
Amphotericin B	8 (30.8)	18 (69.2)	\
Fluconazole	13 (31)	29 (69)	\
Voriconazole	0 (0)	1 (100)	\
Micafungin	26 (40.6)	38 (59.4)	\
Antifungal combination	2 (50)	2 (50)	\
The antifungal has been modified?	\	\	0.255
Yes	3 (20)	12 (80)	\
No	46 (38.3)	74 (61.7)	\
Outcome within 30 days	\	\	0.205
Death	15 (45.5)	18 (54.5)	\
Survival	34 (31.5)	74 (68.5)	\
IC-related death	\	\	0.722
Yes	11 (47.8)	12 (52.2)	\
No	4 (40)	6 (60)	\

ICU, Intensive care unit; CVC, central venous catheter; IC, invasive candidiasis. * Pearson's chi-square test or Fisher's exact test was used. Significant variables (p ≤ 0.05) are indicated in bold.

### Identification and biofilm formation

All *Candida* spp. clinical isolates were stored in skim milk at −80°C until use. They were cultured on Sabouraud Dextrose Agar to assess viability and purity, and identified by MALDI-TOF MS (Microflex LT Biotyper 3.0, Bruker Daltonics, Bremen, Germany) according to the manufacturer’s instructions. Biofilm formation was evaluated as previously described [10].

### Antifungal susceptibility testing

Antifungal susceptibility testing was carried out using the broth microdilution technique for fluconazole (0.125-64 µg/mL), voriconazole (0.016-1.0 µg/mL), amphotericin B (0.063-8.0 µg/mL), and micafungin (0.0078-8.0 µg/mL) according to the European Committee for Antimicrobial Susceptibility Testing (EUCAST). To ensure test quality, two reference strains, *C. parapsilosis* ATCC 22019 and *C. krusei* ATCC 6258, were included as controls for each antifungal susceptibility test. The minimum-inhibitory-concentration (MIC) results were interpreted according to the EUCAST breakpoints [11].

### Genomic DNA extraction and multilocus sequence typing (MLST)

Genomic DNA was extracted using a previously described in-house protocol [12]. MLST was performed for *C. albicans* and *C. tropicalis*. Of 43 *C. albicans* isolates, 42 were viable and typed using seven housekeeping genes (PubMLST database; accessed 10 Feb 2025). Twenty-four of 25 *C. tropicalis* isolates were typed using six housekeeping genes (species database; accessed 10 Feb 2025).

PCR was conducted as previously described [13,14]. Amplicons were analyzed by 1.5% agarose gel electrophoresis, purified with ExoSAP-IT, and sequenced on an ABI 3500 (Applied Biosystems, USA) using the same primers. Electropherograms, allele assignment, and diploid sequence type (DST) determination were performed as previously described [2].

### Microsatellite typing

The microsatellite technique was used to genotype all the isolates of *C. parapsilosis* stricto sensu by amplifying eight polymorphic microsatellite markers [15]. The reference *C. parapsilosis* ATCC 22019 was used to compare the allelic profiles of the isolates. Determination of the allelic profiles and analysis of the clonal isolates were performed as previously described [10].

## Results

### Description of the study population

A total of 141 candidemia episodes were included. The study population comprised predominantly young children with a median age of 1 year (range, 0–17 years). Specifically, 80.2% (n = 113) were aged 0–5 years, and 40.4% (n = 57) were infants younger than 13 months. Detailed baseline characteristics are shown in Table 1.

Demographic characteristics and hospitalization units were similar between the PC and non-PC groups. However, significant risk factors for PC included CVC non-removal within 5 days of treatment initiation (p = 0.005), parenteral nutrition use (p = 0.033), and hematologic or solid malignancies (p = 0.042). Neurological disease was more frequent in the non-PC group (p = 0.02) (Table 1).

In an exploratory analysis of patients whose catheters were removed within 5 days (n = 64), logistic regression showed no significant association between each additional day of catheter retention and persistent candidemia (p = 0.34). Instead, failure to remove the catheter within 5 days was the main factor associated with persistence.

Antifungal therapy was initiated within 5 days of the positive blood culture in 137 of 141 patients (97.2%). Three patients (2.1%) did not receive treatment because they died on the day of blood culture collection, and one patient (0.7%) started therapy after 8 days. Among treated patients (n = 137), 119 (86.9%) received an adequate antifungal dose. Patients with persistent candidemia required a significantly longer treatment duration compared to those without persistence (p = 0.00015).

Regarding the clinical outcomes of the patients, the overall mortality at 30 days was 23.4% (n = 33), and the candidemia-related mortality was 16.3% (n = 23).

In addition to the significant factors in the univariate analysis, the results of the multivariate analysis assessing independent predictors of persistent candidemia are presented in Table 2.

**Table 2:** Multivariate analysis for independent factors associated with persistent candidemia in pediatric patients.

**Variables**	**Estimate**	**p-value**	**Odds_ratio**	**LL**	**UL**
(Intercept)	-1.94	< 0.001	0.14	0.06	0.30
Hematological neoplasia/Solid tumor	1.376	0.013	3.96	1.36	12.2
Non-removal of CVC within (5 days after initiation of treatment)	1.22	0.004	3.39	1.50	7.95
Total parenteral nutrition	1.321	0.004	3.75	1.56	9.42

CVC, central venous catheter; LL, lower limit; UL, upper limit.

### Epidemiology

*C. parapsilosis stricto sensu*(n = 52, 39%) was the most prevalent species in the candidemia cases studied, followed by *C. albicans* (n = 43, 30.5%) and *C. tropicalis* (n = 25, 17.7%). Other candidemia-causing species identified in this study included *C. lusitaniae* (n = 5), *Wickerhamomyces anomalus* (n = 4), *Pichia kudriavzevii* (n = 2), *C. orthopsilosis* (n = 2), *C. famata* (n = 2), *C. guilliermondii* (n = 1)*, Nakaseomyces glabratus* (n = 1), *C. haemulonii* (n = 1), *C. dubliniensis* (n = 1), *C. metapsilosis* (n = 1), and *C. utilis* (n = 1).

Regarding the 49 isolates identified in patients with PC, the distribution of the prevalent isolates remained the same: *C. parapsilosis* stricto sensu (n = 20, 40.8%), *C. albicans* (n = 12, 24.5%), and *C. tropicalis* (n = 9, 18.4%). Other species included *C. lusitaniae* (n = 2), *W. anomalus* (n = 2), *Pichia kudriavzevii* (n = 1), *C. guilliermondii* (n = 1), *C. metapsilosis* (n = 1), and *C. utilis* (n = 1).

There were no significant differences between patients with PC and without PC regarding *Candida* species.

### Microbiological characteristics

Among the 141 isolates, 137 were included in the biofilm assay, as four were not viable for testing. Biofilms were produced by 136 (99.3%) of 137 isolates tested. Of these, 108 (78.8%) were classified as strong biofilm producers (Supplementary Fig. 2).

In total, 124 isolates, including *C. parapsilosis* stricto sensu, *C. albicans, C. tropicalis, P. kudriavzeveii,* and *N. glabratus,* were tested for antifungal sensitivity using EUCAST species-specific clinical breakpoints. The remaining isolates were excluded from susceptibility analysis due to the lack of clinical breakpoints for interpretation. Except for one isolate of *C. tropicalis* resistant to fluconazole (MIC = 8 μg/mL), all the others were sensitive to the antifungals tested. Supplementary Table 1 shows the MIC50 and MIC90 values of the four antifungal drugs tested against the most important species enrolled in this study.

A total of 42 *C. albicans* isolates were viable for molecular typing. According to the PubMLST database, all *C. albicans* isolates presented with previously unreported DSTs, and no isolates with the same allelic profile were found. Regarding *C. tropicalis*, 24 isolates were tested, 18 were classified as new and unique DSTs, and another six isolates were classified as belonging to previously described DSTs: 7, 124, 232, and 238. DST124 was detected in more than one isolate, indicating the presence of clonal profiles (Supplementary Tables 2 and 3).

**Figure 1:**
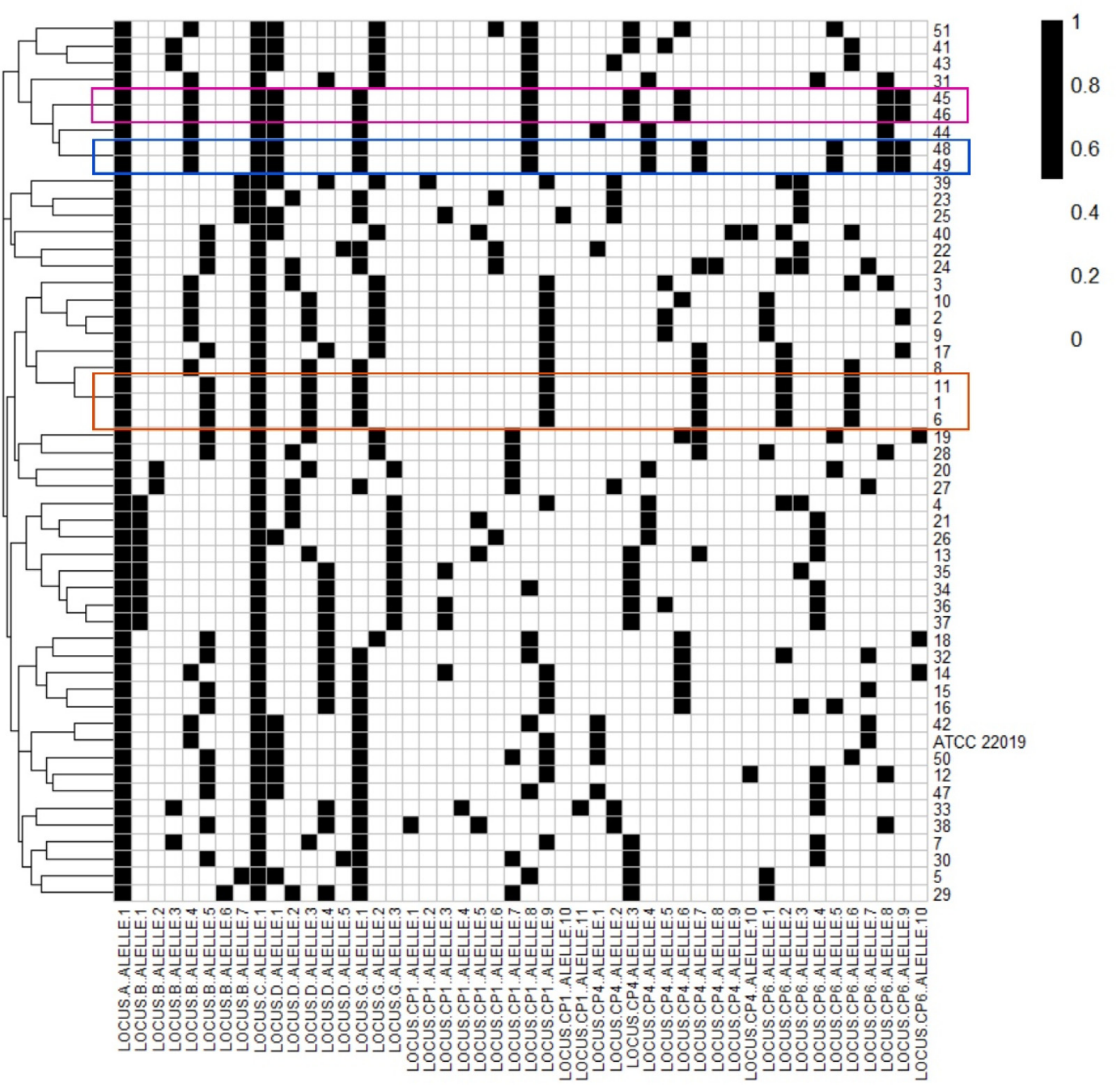
Allelic profile of *C. parapsilosis* stricto sensu isolates grouped in dendogram by genetic similarity. The lines indicate the number (id) of isolates. The black square indicates the presence of an amplification product. The columns correspond to an allele of the respective polymorphic microsatellite marker (8 gene loci). Isolates with the same allelic profile (clones) are highlighted.

**Figure 2:**
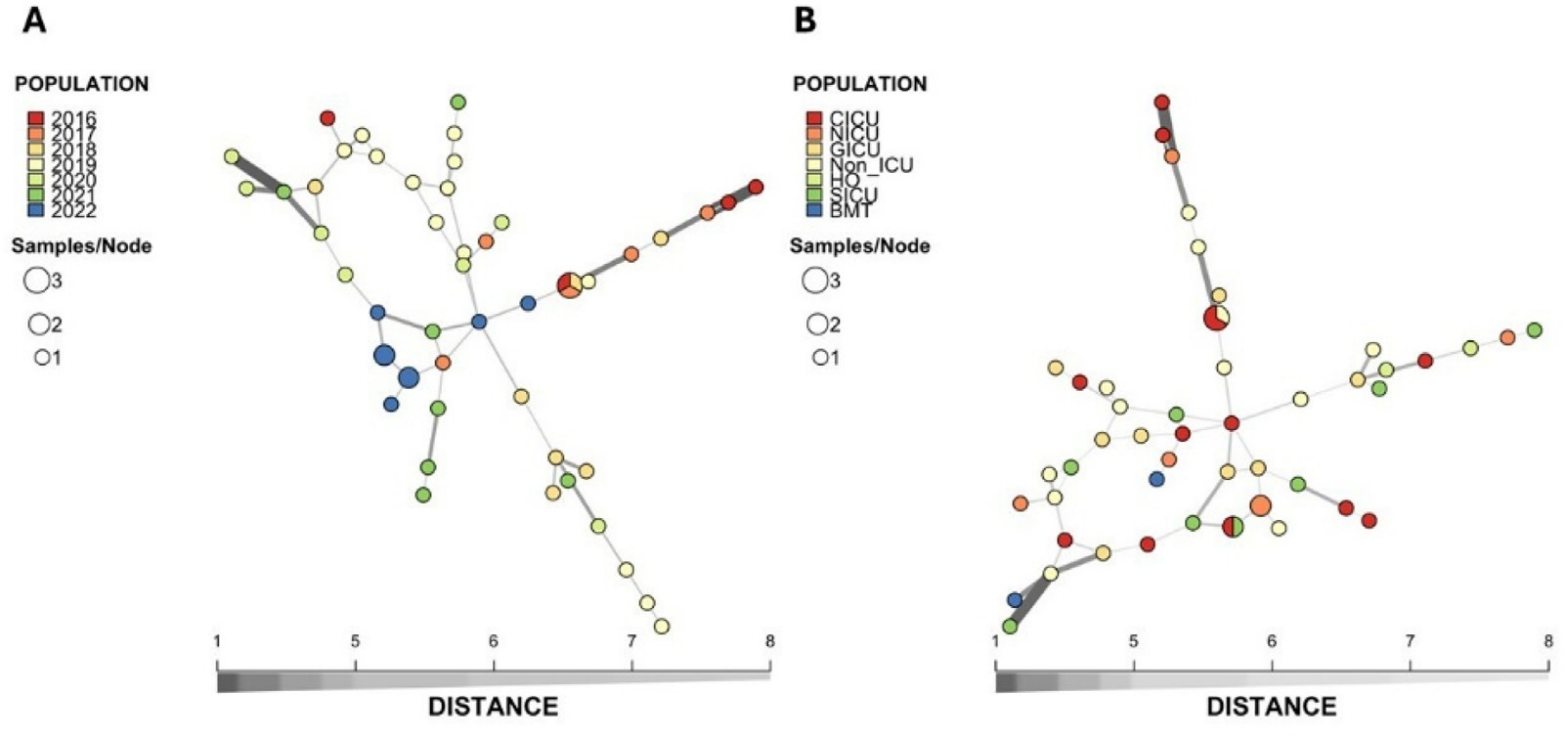
Minimum spanning tree of the relationship between the 47 genotypes among the 51 isolates of *C. parapsilosis* sensu stricto isolated from the bloodstream. Each circle represents a unique genotype, while circle size corresponds to the number of isolates of the specific genotype and the color of the isolates represents the year of isolation (A) or hospital setting. ICU, intensive care unit; CICU, cardiac intensive care unit; NICU, neonatal intensive care unit; GICU, general intensive care unit; Non-ICU, Other clinical units; HO, hematology-oncology; SICU, surgical intensive care unit; BMT, bone marrow transplant.

*C. parapsilosis* sensu stricto isolates were typed using microsatellites to assess genetic similarity. Of 51 isolates, 47 distinct non-clonal genotypes were identified, while seven clonal isolates formed three clusters. Figure 1 shows allelic profiles, including *C. parapsilosis* ATCC 22019, and the distribution of clonal isolates. Genetic relationships among the 47 genotypes, their hospital distribution, and year of isolation are depicted in a minimum spanning tree (Figure 2).

## Discussion

The incidence of PC varies by region, population, and the criteria used to define it. Currently, the absence of a standardized definition hinders accurate estimates, assessment of clinical relevance, and comparability across studies [5,6]. In our study, the incidence of PC was 34.8% (n = 49), considering the criterion of positive blood cultures for a period of five days or more after starting treatment. The observed PC incidence was higher than that in adult patients at 13.7% [7]. These findings reinforce what is described in the literature regarding PC being more frequent in pediatric patients [4,5].

PC has been attributed to host-related aspects, such as oncohematological diseases, fluconazole prophylaxis, prior surgery, abdominal conditions, and the use of invasive devices [6,7]. CVCs contribute to persistence through biofilm formation, making timely removal essential [5,6]. In our study, 91.5% of patients (n = 129) had a CVC, and early non-removal was a significant risk factor for PC (p = 0.005) (Table 1). Consistent with this, CVC removal within 3 days of the first positive blood culture has been linked to shorter candidemia duration (p = 0.002) [16]. To our knowledge, this is the first study to evaluate early CVC removal as a factor in preventing PC across different pediatric age groups.

Although CVC removal in candidemia is recommended by clinical guidelines, it should be noted that CVC removal depends on the patient's clinical stability and the availability of alternative routes for medication administration and laboratory sampling [17]. While we did not assess patient severity or other clinical factors that might influence catheter management, these findings highlight the importance of timely catheter management to reduce PC risk in pediatric patients, while emphasizing individualized clinical decision-making.

Another host factor found to be significant for PC in our study was cancer (p = 0.042) (Table 1). In contrast, Agnelli et al. (2019) found no difference between PC and non-PC groups in relation to this variable, although there was a higher proportion of solid tumors in patients with PC (p = 0.095). The use of NPT was also a significant factor in our sample (p = 0.033) (Table 1). In turn, a previous study with neonates found that only two patients did not undergo NPT, which was not a significant factor for PC in this population [18]. In addition, patients with neurological diseases tended to be in the non-PC group (p = 0.02) in the univariate analysis. However, multivariate analysis revealed that this was not a significant factor, as demonstrated by Agnelli et al. (2019).

Although antifungal choice was not a significant factor, PC patients required longer treatment than non-PC patients (p < 0.00015). Prolonged antifungal use directly impacts patients by increasing the risk of adverse effects, toxicity, antimicrobial resistance, and healthcare-associated infections, while also raising treatment costs [19].

*C. parapsilosis* stricto sensu was the prevalent species, even among patients with PC. Consistent with our findings, *C. parapsilosis* stricto sensu has become predominant in the pediatric population, especially in neonatal ICUs and patients with PC [10,18]. We observed no association between the different species of *Candida* and persistence of the infection.

No association was observed between the ability to produce biofilm and persistent candidemia, possibly due to the high prevalence of biofilm-forming isolates. In our study, except for one isolate of *C. parapsilosis* stricto sensu, all others produced biofilms with 78.8% (n = 108) considered strong producers. In agreement with this, a study conducted between 2010 and 2018 in a tertiary hospital in Spain did not observe biofilm production as a risk factor for PC [7].

Antifungal resistance in *Candida* spp., including azole-resistant *C. tropicalis* and echinocandin- and azole-resistant *C. parapsilosis*, raises public health concerns due to increasing prevalence. However, resistance does not appear to be associated with PC [18]. In this study, the prevalence of resistance was low. Only 1 isolate of *C. tropicalis* was resistant to fluconazole (MIC = 8 µg/mL), and the others were sensitive to the drugs tested, which does not explain the incidence of PC in our institution. This previously described isolate harbored a Y257H mutation in the *ERG*11 gene [20].

Fifty-one *C. parapsilosis* stricto sensu isolates were typed using microsatellites, identifying 47 distinct genotypes and a low prevalence of clonal isolates, suggesting a multifactorial origin rather than cross-contamination [10]. Previous studies suggest that C. parapsilosis associated with CVC may originate from skin contamination near the insertion site, transient colonization of healthcare workers’ hands, or translocation from the patient’s intestinal microbiota, which may contribute to the high genetic diversity observed in this study [[21], [22], [23]].

MLST, based on six to eight housekeeping genes, provides reproducible and highly accurate data [24]. All *C. albicans* isolates belonged to DSTs not previously described in PubMLST, with no clonal isolates identified, indicating high genetic diversity, consistent with previous reports [25]. Among 24 *C. tropicalis* isolates, six matched previously described DSTs (7, 124, 232, 238), while 18 were novel. DST124 was found in three clonal isolates (Ctr25, Ctr27, Ctr48), with Ctr25 and Ctr27 recovered from the same period, unit (hemato-oncology), and patients with PC.

Candidemia is a severe condition associated with high mortality rates, which can exceed 50% of cases, although it tends to be lower in the pediatric population [3,8]. Previously, at our institution, the mortality rate of pediatric patients with candidemia was 32% [3]. In our study, we observed overall 30-day mortality and PC related mortality rates of 23.4% (n = 33) and 16.3% (n = 23), respectively. Hammoud et al. (2013) reported that PC markedly increased mortality, as more than 50% of neonates with this complication died, compared to only 3% of non-PC neonates. In our study, there were no significant differences in related mortality between the PC and non-PC groups (p = 0.722), which is similar to the findings of other studies [5,7]. It should be noted that we did not analyze the risk factors for mortality in patients with PC in our study. Instead, we investigated whether PC was a predictive factor of increased mortality in patients with this complication.

Our study had some limitations, mainly due to its retrospective and single-center design. Multicenter studies evaluating the impact of persistence on length of stay, morbidity, and hospital costs are important.

In conclusion, this study showed a high incidence of PC in pediatric patients and its association with host factors and clinical management of the patient, while the characteristics of the etiological agent were not risk factors for this complication. Furthermore, PC was not related to increased mortality but led to a prolonged treatment period, which could result in increased hospital costs and additional risks for the patient.

## Funding

This study was financed in part by the Coordenação de Aperfeiçoamento de Pessoal de Nível Superior, Brazil (CAPES) - Finance code 001.

## Data availability statement

The data that support the findings of this study are available from the corresponding author.

## Conflicts of interest

The authors declare no conflicts of interest.

## Appendix Supplementary materials

Image, application 1
