# Supplementary material for: Persistent candidemia in pediatrics: exploring risk factors
Source: J Pediatr (Rio J). 2026 Mar 25;102(3):101535. doi: 10.1016/j.jped.2026.101535 (PMC13052017; doi:10.1016/j.jped.2026.101535)

**JPED-D-25-00412_Supplementary Material**

**Supplementary Table 1** Minimum inhibitory concentrations (MIC50 and MIC90) of the prevalent species in the study.

| **Species** | **Amphotericin B** | | | | **Fluconazole** | | | | **Voriconazole** | | | | **Micafungin** | | | | |
| --- | --- | --- | --- | --- | --- | --- | --- | --- | --- | --- | --- | --- | --- | --- | --- | --- | --- |
|  | MIC_50_ | MIC_90_ | S ≤ | R > | MIC_50_ | MIC_90_ | S ≤ | R > | MIC_50_ | MIC_90_ | S ≤ | R > | MIC_50_ | | MIC_90_ | S ≤ | R > |
| *C. parapsilosis stricto sensu* | ≤0,06 | ≤0,06 | 1 | 1 | 0,5 | 1 | 2 | 4 | ≤0,016 | 0,03 | 0,13 | 0,25 | 0,5 | 1 | | 2 | 2 |
| *C. albicans* | 0,125 | 0,125 | 1 | 1 | 0,25 | 0,5 | 2 | 4 | ≤0,016 | ≤0,016 | 0,06 | 0,25 | ≤0,007 | ≤0,007 | | 0,02 | 0,02 |
| *C. tropicalis* | ≤0,06 | 0,125 | 1 | 1 | 0,5 | 1 | 2 | 4 | ≤0,016 | 0,03 | 0,13 | 0,25 | 0,015 | 0,015 | | IE^5^ | IE^5^ |

MIC, minimum inhibitory concentration; MIC_50_, represents the MIC value at which ≥ 50% of the isolates tested are inhibited; MIC_90_, represents the MIC value at which ≥ 90% of the isolates tested are inhibited; S, sensitive; R, resistant; IE, no clinical breakpoint.

**Supplementary Table 2** Molecular typing of *C. albicans* isolates using MLST.

| **ID** | ***C. albicans –* housekeeping genes** | | | | | | | **DST** |
| --- | --- | --- | --- | --- | --- | --- | --- | --- |
|  | ***AAT1a*** | ***ACC1*** | ***ADP1*** | ***MPIb*** | ***SYA1*** | ***VPS13*** | ***ZWF1b*** |  |
| Cal1 | new | new | new | new | new | 108 | new | new |
| Cal10 | 13 | 28 | 15 | new | new | 160 | new | new |
| Cal12 | 115 | new | new | new | new | 45 | new | new |
| Cal14 | 8 | new | new | new | 2 | 6 | 5 | new |
| Cal21 | 13 | 7 | 10 | new | new | 55 | 29 | new |
| Cal25 | 13 | 10 | new | new | new | new | 29 | new |
| Cal29 | 13 | new | new | 4 | new | 37 | new | new |
| Cal31 | 13 | new | new | new | new | 32 | 15 | new |
| Cal32 | 13 | new | new | 6 | new | 37 | 15 | new |
| Cal34 | new | new | new | new | 34 | 26 | 4 | new |
| Cal44 | 115 | new | new | new | new | 45 | 15 | new |
| Cal49 | new | 2 | new | new | 2 | 6 | new | new |
| Cal51 | 115 | new | new | new | new | 45 | new | new |
| Cal52 | 2 | new | 2 | new | 2 | 6 | 300 | new |
| Cal54 | 13 | new | new | 6 | new | 37 | 22 | new |
| Cal55 | new | 3 | new | 9 | 2 | 6 | 5 | new |
| Cal66 | 13 | new | new | new | new | new | 15 | new |
| Cal68 | 8 | new | new | 4 | new | 10 | 8 | new |
| Cal73 | 13 | new | new | 4 | new | 32 | 22 | new |
| Cal74 | 8 | new | new | 4 | new | 10 | 8 | new |
| Cal75 | new | new | 6 | new | 2 | 20 | 5 | new |
| Cal77 | 13 | new | new | new | new | new | 40 | new |
| Cal78 | new | new | new | 4 | new | 10 | 8 | new |
| Cal79 | new | new | new | 9 | 2 | new | 5 | new |
| Cal82 | new | 3 | new | new | new | 16 | 95 | new |
| Cal83 | 8 | 14 | new | 4 | new | 10 | 22 | new |
| Cal90 | new | 5 | new | 9 | 2 | new | 5 | new |
| Cal91 | 2 | new | 6 | new | 2 | new | 5 | new |
| Cal93 | 3 | 2 | new | new | 2 | new | 83 | new |
| Cal95 | new | new | new | 9 | 2 | new | 30 | new |
| Cal101 | 13 | 13 | new | new | new | 37 | 137 | new |
| Cal107 | 2 | 3 | 5 | 9 | 2 | new | 339 | new |
| Cal115 | 115 | 134 | 4 | 198 | 272 | 45 | 29 | new |
| Cal117 | new | 7 | 6 | 14 | new | 45 | 236 | new |
| Cal118 | 210 | 129 | 5 | 9 | 2 | 25 | 339 | new |
| Cal121 | 13 | 7 | 12 | new | new | 110 | 92 | new |
| Cal122 | 5 | 3 | 2 | 9 | 2 | new | 25 | new |
| Cal124 | 210 | 129 | 2 | 197 | 2 | 6 | 52 | new |
| Cal126 | 210 | 5 | 5 | 197 | 2 | new | 5 | new |
| Cal127 | 13 | 7 | 14 | 193 | 2 | new | 15 | new |
| Cal129 | 8 | 5 | 5 | 197 | 2 | 68 | 5 | new |
| Cal132 | 13 | 7 | 14 | 193 | 270 | 37 | 15 | new |

ID, isolate identification; DST, diploid sequence type.

**Supplementary Table 3** Molecular typing of *C. tropicalis* isolates using MLST.

| **ID** | ***C. tropicalis –* housekeeping genes** | | | | | | **DST** |
| --- | --- | --- | --- | --- | --- | --- | --- |
|  | ***ICL1*** | ***MDR1*** | ***SAPT2*** | ***SAPT4*** | ***XYR1*** | ***ZWF1a*** |  |
| Ctr1 | new | 32 | 4 | new | 77 | 10 | new |
| Ctr2 | 23 | 158 | 3 | 13 | 185 | 4 | new |
| Ctr3 | 1 | 7 | 4 | 9 | 50 | 1 | new |
| Ctr4 | 1 | 7 | 3 | 10 | 4 | 6 | new |
| Ctr11 | 5 | 53 | 4 | 9 | 92 | 9 | new |
| Ctr13 | 1 | 1 | 18 | 1 | 19 | 1 | new |
| Ctr24 | 1 | 17 | 2 | 14 | 2 | 3 | new |
| **Ctr25** | **1** | **24** | **3** | **7** | **48** | **6** | **124** |
| **Ctr27** | **1** | **24** | **3** | **7** | **48** | **6** | **124** |
| Ctr40 | 1 | 39 | 3 | 7 | new | 17 | new |
| Ctr46 | 1 | 7 | 4 | 6 | 52 | 4 | 238 |
| **Ctr48** | **1** | **24** | **3** | **7** | **48** | **6** | **124** |
| Ctr53 | new | 218 | 3 | 7 | new | new | new |
| Ctr58 | 1 | 7 | 3 | 6 | 192 | 10 | new |
| Ctr60 | 3 | 7 | 3 | 6 | 6 | 4 | 7 |
| Ctr61 | 1 | 24 | 3 | 7 | 24 | 6 | 232 |
| Ctr68 | 1 | 1 | 3 | new | new | 1 | new |
| Ctr70 | 3 | 4 | 3 | new | 77 | 4 | new |
| Ctr71 | 5 | new | 1 | new | 92 | 9 | new |
| Ctr72 | 42 | 1 | 18 | 1 | 5 | 1 | new |
| Ctr74 | 1 | 1 | 3 | 1 | 185 | new | new |
| Ctr77 | 1 | 17 | 2 | 14 | 9 | 3 | new |
| Ctr78 | new | 4 | 3 | new | 77 | new | new |
| Ctr80 | new | 143 | 3 | new | new | new | new |

ID, isolate identification; DST, diploid sequence type. Clonal isolates are bolded.

**Supplementary Figure 1** Flowchart of candidemia cases selection.


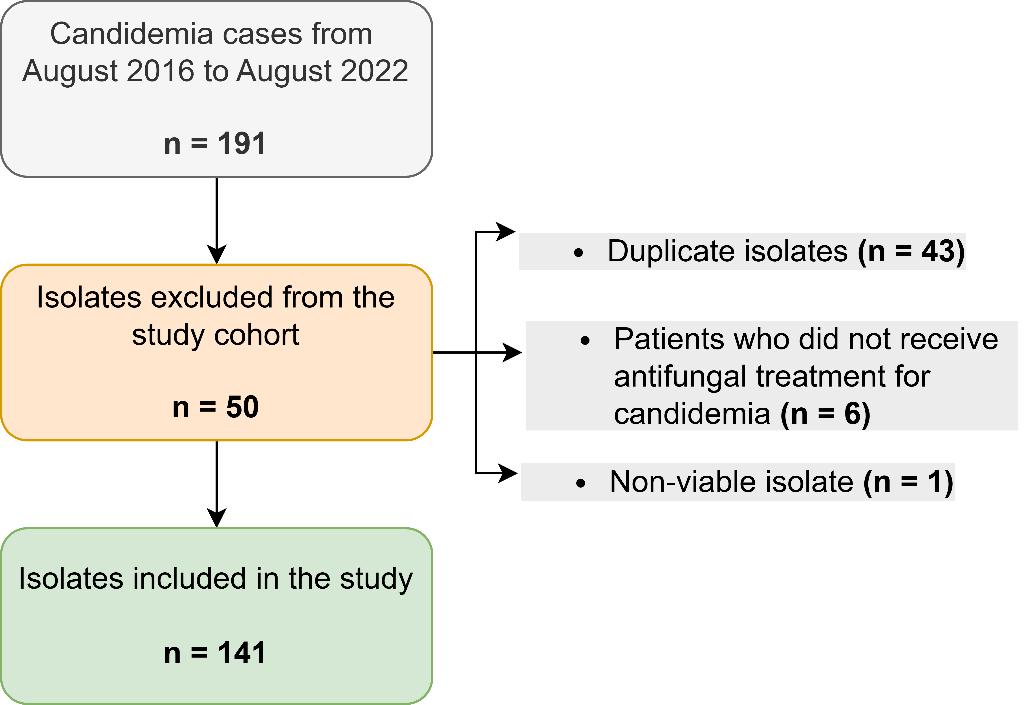


**Supplementary Figure 2** Biofilm production capacity of *Candida* species.


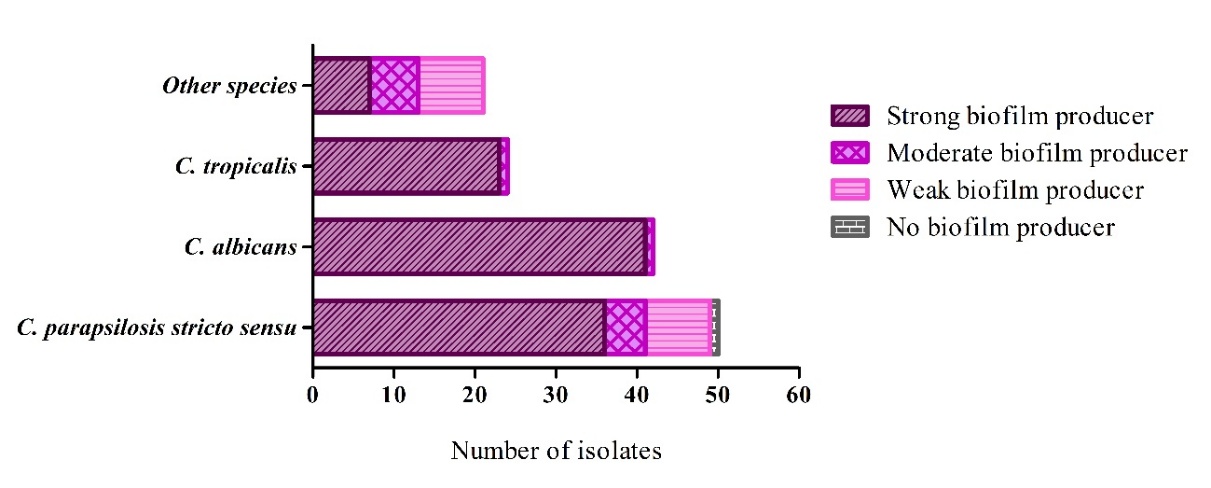

Supplement: Supplementary file 1 [file mmc1.docx]
